# Levels of HIV/AIDS stigma and associated factors among sexually active Ethiopians: analysis of 2016 Ethiopian Demographic and Health Survey Data

**DOI:** 10.1186/s12889-022-13505-1

**Published:** 2022-05-31

**Authors:** Merga Belina Feyasa, Mamo Nigatu Gebre, Teshome Kabeta Dadi

**Affiliations:** 1 Department of Statistics, College of Natural & Computational Sciences, Addis Ababa University, Addis Ababa, Ethiopia; 2 Department of Epidemiology, Institute of Health, Faculty of Public Health, Jimma University, Oromia Jimma, Ethiopia

**Keywords:** EDHS, Ethiopia, HIV-related stigma, PLWHA

## Abstract

**Background:**

Stigma and discrimination have fueled the transmission of the disease and dramatically increased its negative public health impact. Even though the disease has extremely ravaged human life, stigma, and discrimination attached to it are not well addressed in Ethiopia at the country level. The reduction of stigma and discrimination in a population are important indicators of the success of programs that target HIV prevention and control. This study aimed to assess the level of HIV-related stigma and its determinants among sexually active Ethiopians.

**Methods:**

A public domain data were obtained from 2016 Ethiopian Demographic and Health Survey in which two-stage cross-sectional stratified cluster sampling was applied. A total of 28,371 sexually active Ethiopians were interviewed from both rural and urban parts of Ethiopia. Descriptive Statistics and multilevel ordinal logistic regression (proportional odds model) were used to summarize data and to investigate correlates of HIV-related stigma.

**Results:**

Only 5.1% (95% CI: 4.5%, 5.8%) of sexually active Ethiopians did not have a stigmatizing attitude, whereas, 59.2% (95% CI: 57.3%, 61.1%) and 35.65% (95% CI: 33.5%, 37.9%) of them had a moderate and high level of stigma respectively. Regression results show that residence (AOR = 1.82, 95% CI:1.46, 2.27), education (AOR = 0.65,95% CI: 0.50,0.84), owning mobile (AOR = 0.63,95% CI:0.55,0.72), HIV-testing (AOR = 0.77, 95% CI:0.70,0.84), age (AOR = 0.81, 95% CI: 0.73, 0.91), religion (AOR = 1.53,95% CI:1.33,1.76), and marital status (AOR = 1.38, 95% CI:1.19, 1.61) were significantly associated with HIV-related stigma (*p* < 0.0001).

**Conclusion:**

Regardless of all efforts put in a place to prevent and control HIV, a significant proportion of sexually active Ethiopians have stigmatizing attitudes. Residence, educational level, owning mobile, HIV test uptake, age, religion, and marital status were determinants of HIV-related stigma. Expanding mobile coverage, promoting HIV counseling and tests, promoting HIV education, and working with religious leaders, among other strategies could be used to minimize the stigma attached to the disease to best prevent and control it.

## Background

HIV-related stigma and discrimination are hindering accessibility to HIV-related services and support programs [1–3]. According to article 23 of the Universal Declaration of Human Rights, everyone has the right to work, to free choice of employment, to just and favorable conditions of work, and to protection against unemployment [4]. Nonetheless, starting from the beginning of the HIV/AIDS pandemic, stigma and discrimination have fueled the transmission of HIV and have greatly increased the negative impact of the disease. Stigma can lead to discrimination and other violations of human rights which affect the well-being of PLWHA fundamentally [5]. The discrimination, extreme suffering, and human rights violations that occurred and continue to occur have reached far beyond the disease itself [6].

Different studies have indicated that a high proportion of PLWHA are being stigmatized and discriminated against by a significant proportion of the population they are sharing lives with even though the severities differ from country to country and from place to place [7–13]. The report from the 2017 Joint United Nations Program on HIV and AIDS (UNAIDS) showed that twenty percent of PLWHA across 19 countries where there were available data had avoided going to a clinic or hospital because of the fear of stigma or discrimination related to their HIV status [14]. A qualitative exploratory study done in Dar Es Salaam, Tanzania, and a cross-sectional study done in Nigeria similarly indicated that HIV-related stigma and discrimination compromised adherence to antiretroviral therapy (ART) by reinforcing the concealment of HIV status and undermining social support [15, 16]. On the other hand, a cross-sectional study from Iran depicted that HIV/AIDS-related stigma decreases the quality of life among PLWHA [17].

The results from different studies have shown that Knowledge about HIV, educational status, age, place of residence, and marital status are some of the common factors associated with stigma and discrimination towards PLWHA [7, 8, 13, 18].

According to 2016 WHO Ethiopian country HIV profile, report, an estimated number of 710,000 people were living with HIV and only 59% of PLWHA were receiving ART [19]. Although more than half a million people are living with HIV and more than forty percent of PLWHA are not taking HIV treatment in the country, HIV-related stigma and discrimination towards PLWHA are not well addressed using nationally representative data. Therefore, the current study is aimed at assessing the magnitude of stigma towards PLWHA and its determinants using the nationally representative 2016 EDHS data.

## Methods

### Data sources

The 2016 EDHS is the fourth (*conducted in 2016*) Demographic and Health Survey (DHS) collecting data and producing reports since 2000 via the Central Statistical Agency (CSA) in Ethiopia. These data were processed and organized by the International Classification of Functioning (ICF) International into different datasets. The authors secured permission to access these public domain datasets from the MEASUREDHS website.

### Study population and sampling procedures

All DHSs follow stratified cluster sampling procedures, the strata are the urban and rural types of places of residence and the clusters are the enumeration area (EAs) which was formed by dividing kebeles in that each EA contains 250 to 300 households before conducting the 2007 census [20]. A cross-sectional data was obtained in which a two-stage sampling design with stratification into urban and rural was employed by sampling 645 EAs, 202 from urban and 443 from rural as the first stage, and selection of 28 households from each sampled EAs by a probability proportionate to the size (PPS) considered as the second stage. Finally, 18,008 households were selected from which 17,067 households were occupied and 12,688 eligible males aged from 15 to 59, and 15,683 eligible women aged from 15 to 49 years were identified and interviewed [21]. In this study, there were no inclusion or exclusion criteria employed. All of the (*n* = 28,371) available participants were included.

### Measurements

HIV stigma levels were determined by the six domains of stigma and discrimination questions [1]. The socio-demographic variables (region, sex, age, religion, wealth status, marital status, occupational status, place of residence, sex of household head, and education level), owning mobile telephone, use of the internet, frequency of using the internet within a month, frequency of reading newspaper in a month, frequency of listening to the radio in a month, frequency of watching television, the status of testing for HIV and health insurance were measured by respective direct questions asked from the respondents. The other two, namely risky sexual behavior and knowledge of HIV were indirectly measured by asking different indicator questions from which scoring and grouping were done to measure each variable separately.

### Operational definitions

The guiding methods of measuring HIV stigma and discrimination were clearly outlined for the general population, PLWHA, and healthcare workers [1]. Since the respondents were all females and males who were sampled from the community, questions designed for the general population were used. Accordingly, six domains of measuring HIV stigma and discrimination were defined below.

#### Fear of infection

A respondent is considered to have a fear of infection if he/she fears contracting HIV if she/he comes into contact with the saliva of a PLWHA.

#### Social judgment

Existence of fear of social judgment if the respondents were ashamed if someone in the family has HIV.

#### Anticipated stigma

Manifested if the respondents answered positively for questions that asked about people being hesitant to take an HIV test due to fear of people’s reaction if the test result is positive for HIV.

#### Perceived stigma

The respondents were considered to have perceived stigma if they thought that people talk badly about people living with or thought to be living with HIV to others or if the respondents thought that people living with or thought to be living with HIV lose respect.

#### Experienced stigma

The experience of discrimination, based on HIV status or association with a person living with HIV or another stigmatized group, that falls outside the purview of the law (Examples of discrimination that fall outside the purview of the law include: blaming, discrediting, gossip, verbal harassment, avoiding everyday contact, ostracism and abandonment). The respondents were grouped under experienced stigma if people didn’t buy fresh vegetables from a shopkeeper or vendor knowing that they had HIV or faced any of the above examples.

#### Discrimination inside legal purview

The respondents who were against the thought that children living with HIV should be able to attend school with children who are HIV negative or those who disagree with the idea that female teacher that has HIV but is not sick, should be allowed to continue teaching in the school.

The summation of these six questions was done and the scores ranging from 0 to 6 were grouped into three classes. ‘No stigma’ if the score of the six domains’ sum is 0, ‘moderate stigma’ level if the sum ranges between 1 and the average (three); and ‘high stigma’ level if the sum is greater than the average and up to the maximum score (four to six).

#### Experiencing sexual behavior

The respondents were grouped as practicing risky sexual behavior if they responded either as having multiple sex partners in a lifetime or if they had multiple sex partners in the last 12 months excluding the spouse.

The variable, ‘knowledge about HIV’ was constructed by combining the responses to the nine sets of questions. Scores were computed from the question that asks whether the respondents ever heard about HIV/AIDS, whether the respondent knows about reducing the risk of getting HIV by always using condoms during sex, knows about reducing the risk of getting HIV by having 1 sex partner only who has no other partners, answered correctly regarding transmissions of HIV via mosquito bites and by sharing food with a person who has AIDS, a healthy-looking person can have HIV, know that HIV can be transmitted during pregnancy, delivery, and breastfeeding. The scores ranging from 0 to 9 were categorized to form the ‘HIV knowledge’ levels of the respondents. The levels of the variable ‘knowledge about HIV’ then recoded as 0 = ‘*No knowledge*’, 1–8 = ‘*inadequate knowledge*’, and 9 = ‘*comprehensive knowledge’* of HIV.

#### Age category

We have three age categories, namely, *youths* are males or females whose ages in 15–29 years old, *adults* are males or females whose ages fall in the age group of 30- 44 and *late adults* are females whose ages range from 45 to 49. According to Ethiopian labor policy youths are defined to be individuals in the age range 15 – 29 [22–24].

The occupational status includes Agricultural workers, professional workers, trade or sales workers, elementary occupation, and other workers.

Sexually active people: includes all men aged 15–59 and all women aged 15–49 per the 2016 EDHS report.

Kebele is the lowest administrative unit in the government administrative structure of the Federal and Democratic Republic of Ethiopia. The administrative structure of the Ethiopian government from highest to lowest is as follows: Federal government, regions (currently 11 regions), Zones (similar to provinces), Woredas (similar to districts), Kebeles (similar to sub-districts).

### Data analysis

Descriptive and inferential analyses were done using Stata 14.2 statistical software. The multilevel ordinal logistic regression model was fitted to assess regional variation of HIV/AIDS stigma level and identify associated factors. The EDHS surveys often follow a hierarchical data structure as the surveys are based on two-stage stratified cluster sampling [21]. Models used for the analysis of hierarchical data structure must account for associations among observations within clusters (levels) to make efficient and valid inferences. When the variance of the residual errors is correlated between individual observations as a result of these nested structures, single ordinal logistic regression is inappropriate, consequently, multilevel ordinal logistic regression (proportional odds model for clustered data) was used to assess the relationship between levels of HIV/AIDS stigma and associated factors.

The multilevel ordinal logistic regression model is given as follows. Let the *K*-ordered response categories be coded as *k* = *1, 2, …, K.* Ordinal response models often utilize cumulative comparisons of the ordinal outcome. The cumulative probabilities for the *K* categories of the ordinal outcome *Y* are defined as

$${P}_{ijk}=P\left({Y}_{ij}\le k\right)=\sum_{m=1}^{k}{p}_{ijm}.$$

The multilevel logistic model for the cumulative probabilities is given in terms of the cumulative logits as

$$\mathrm{log}\left(\frac{{p}_{ijk}}{1-{p}_{ijk}}\right)={\delta }_{k}-\left[{X}_{ij}^{^{\prime}}\beta +{{Z}^{^{\prime}}}_{ij}T{\theta }_{j}\right], k=1,\dots , K-1,$$

with K − 1 strictly increasing model thresholds ${\delta }_{k}$ (i.e., ${\delta }_{1}$ < ${\delta }_{2}$... < ${\delta }_{K-1}$).

In classical regression, estimates of varying effects can be noisy, especially when there are few observations per group; multilevel modeling allows us to estimate these interactions to the extent supported by the data. In multilevel regression, the clustering effect plays a great role in the estimation of the parameters and this clustering effect can be quantified by intraclass correlation (ICC). ICC is the proportion of total variation in the response variable that is accounted for by between-group variation [25]. The intra-class correlation (ICC) shows the proportion of total variance that is explained by cluster-level (i.e., level 2: region) and is given by

$$ICC=\frac{{\sigma }_{region}^{2}}{{\sigma }_{region}^{2}+{\sigma }^{2}}$$

, where ${\sigma }_{region}^{2}$ is the cluster or level-2 variance and *σ*^2^ is the level-1 variance.

In the current study, all predictors are at level 1 and the authors are interested in studying the effect of the clustering variable, which is a region where the subjects were dwelling. On the other hand, the categories of the response variable (levels of stigma) are three, which are ordered as ‘no’, ‘moderate’ and ‘high’ stigma and hence order logistic regression has potentially greater power than that of binary logistic regression and the baseline-category logit models as it takes into account information on the order of values [26].

All the outputs for descriptive analysis were done using weights provided in EDHS 2016 data as per the recommendations by the DHS program. The weights from EDHS were adjusted and used to carry out multilevel analysis as per the recommendation by Adam [27]. Subsequently, we have checked the goodness of fit after weighting the dataset by both candidate weights. The regression fitted by using the adjusted weights resulted in lower AIC = 34,631.82, and BIC = 34,721.89 as compared to the results from unadjusted weights with AIC = 39,417.69 and BIC = 39,548.7. In addition to the choice of weights, the principle of parsimony dictates us to go for the model with fewer numbers of variables in the model. Consequently, significant variables retained in our final model are presented in Table 1.

**Table 1:** Result of multilevel ordinal logistic regression for predictors of stigma among sexually active men and women in Ethiopia, EDHS 2016

Predictors	Odds Ratio	Robust Std. Err	z	P > z	[95% Conf. Int.]	
**Residence (Ref.** Urban**)**						
Rural	1.82	0.204	5.34	< 0.001	1.46	2.27
**Educational level (Ref.** No education**)**						
Primary	0.65	0.084	-3.33	< 0.001	0.5	0.84
Secondary	0.42	0.06	-6.07	< 0.001	0.32	0.56
Higher	0.43	0.05	-7.3	< 0.001	0.35	0.54
**Owns Mobile (Ref.** No**)**						
Yes	0.63	0.043	-6.88	< 0.001	0.55	0.72
**Ever tested for HIV (Ref.** No**)**						
Yes	0.77	0.038	-5.38	< 0.001	0.7	0.84
**Age (Ref.** Youth**)**						
Adult	0.93	0.042	-1.73	0.084	0.84	1.01
Late Adult	0.81	0.046	-3.61	< 0.001	0.73	0.91
**Religion (Ref.** Orthodox**)**						
Protestant	1.53	0.108	5.97	< 0.001	1.33	1.76
Muslim	1.08	0.104	0.8	0.422	0.89	1.3
Other	1.55	0.272	2.51	0.012	1.1	2.19
**Marital Status (Ref.** Never in a union**)**						
Married	1.38	0.107	4.17	< 0.001	1.19	1.61
Other	1.15	0.105	1.53	0.127	0.96	1.38
Intercept 1	-3.22	0.252	-12.76	< 0.001	-3.71	-2.72
Intercept 2	0.9	0.217	4.13	< 0.001	0.47	1.32
**Region**						
var(_cons)	0.14	0.046			0.07	0.26

## Results

### Characteristics of the participants

**Fig. 1:**
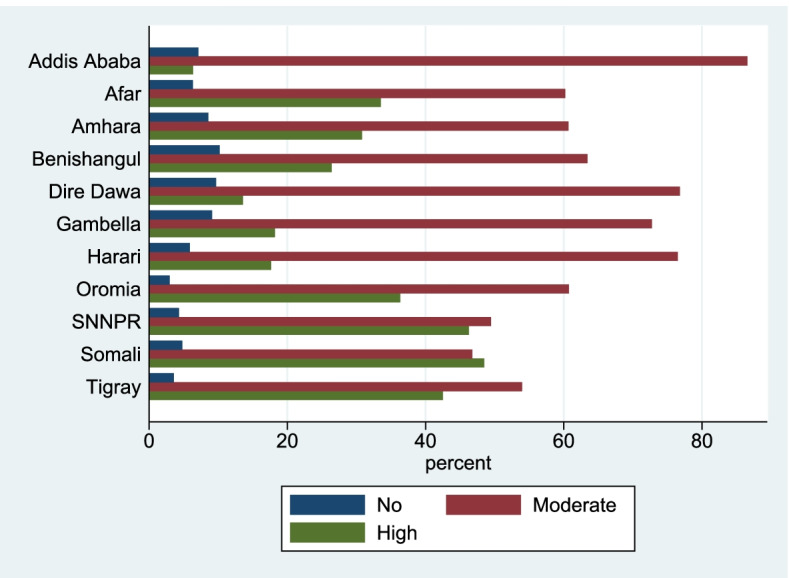
Prevalence of HIV Stigma levels among sexually active men and women by regions in Ethiopia, 2016 EDHS. Note on proportional odds assumption. We followed the procedure of Donald Hedeker [28]. To test the proportional odds assumption, we proceed by additionally estimating a model that relaxes this assumption. The idea behind this is that we compare the model that assumes proportional odds to the model that relaxes this assumption. If the latter fits the data (statistically) better, then the assumption of proportional odds is rejected. Comparing the deviance statistics, we obtain a likelihood-ratio. LR chi2(1) = -23,932.03; Prob > chi2 = 1.0000, which is not statistically significant. Thus, the proportional odds assumption is not rejected for these data

According to the 2016 EDHS, only about 19.9% of sexually active Ethiopians attended beyond primary school, whereas, about 40% didn’t attend school. The majority of the respondents, about 79%, are rural residents. About 80% of the respondents are from male-headed households. Nearly 41% of the respondents are engaged in agricultural works. About 11% of respondents are late adults (45–59), whereas the majority, about 55% of them are youth (15–29). Most of the respondents (44%) are followers of the Orthodox religion followed by protestant religion followers (22.6%). Almost three-fifth (60.1%) of the respondents were married (Table 2). The distribution of the prevalence of each level of stigma manifestation of sexually active Ethiopians by region is presented in Fig. 1. Nearly 5% of the participants do not know about HIV at all while 91.9% and 3.2% of the participants have inadequate and comprehensive knowledge of HIV respectively. The descriptive result depicts that Addis Ababa is leading by the prevalence of manifesting a moderate level of stigma towards PLWHA.

**Table 2:** Socio-demographic and economic characteristics of sexually active men and women in Ethiopia, EDHS 2016

**Items**	**Category**			**Stigma Level**					
		**Total**		**No**		**Moderate**		**High**	
		**N**	**%**	**N**	**%**	**N**	**%**	**N**	**%**
**Educational level**	No education	11,338	40.0	329	3.2	4,963	48.0	5,048	48.8
	Primary	1,392	40.2	594	5.4	6,487	58.7	3,968	35.9
	Secondary	3,664	12.9	328	9.0	2,830	77.5	494	13.5
	Higher	1,978	7.0	134	6.8	1,715	87.1	121	6.1
**Residence**	Urban	5,977	21.1	472	8.0	4,664	79.0	768	13.0
	Rural	2,394	78.9	912	4.3	11,331	53.7	8,862	42.0
**Sex of household head**	Male	3,083	81.4	1,075	4.9	12,801	58.2	8,109	36.9
	Female	5,288	18.6	309	6.2	3,195	63.6	1,521	30.3
**Occupational Status**	Not working & didn't work in last 12	8,770	30.9	426	5.3	4,527	56.1	3,119	38.6
	Agricultural Workers	1,681	41.2	490	4.3	6,200	55.0	4,580	40.6
	Professional Workers	1,215	4.3	82	6.8	996	82.8	124	10.3
	Trade/Sales	3,224	11.4	178	5.6	2,006	63.6	971	30.8
	Elementary occupation	2,000	7.0	121	6.3	1,314	68.6	481	25.1
	Others	1,482	5.2	88	6.3	954	68.3	355	25.4
**Age**	Youth	15,531	54.7	856	5.8	9,151	62.1	4,736	32.1
	Adult	9,793	34.5	400	4.3	5,195	55.6	3,749	40.1
	Late Adult	3,047	10.7	129	4.4	1,650	56.4	1,145	39.2
**Wealth Status**	Poor	9,769	34.4	283	3.2	4,397	49.2	4,262	47.7
	Middle	5,421	19.1	237	4.6	2,721	52.9	2,184	42.5
	Rich	13,181	46.5	864	6.7	8,878	68.7	3,184	24.6
**Religion**	Orthodox	12,476	44.0	803	6.6	7,711	63.4	3,642	30.0
	Protestant	6,422	22.6	203	3.3	3,254	52.6	2,724	44.1
	Muslim	8,878	31.3	353	4.3	4,797	59.0	2,979	36.6
	Other	595	2.10	26	4.7	234	42.9	286	52.4
**Marital Status**	Never in union	8,931	31.5	624	7.3	5,771	67.8	2,121	24.9
	Married	17,062	60.1	678	4.2	8,754	53.8	6,836	42.0
	Other	2,378	8.4	83	3.7	1,471	66.0	673	30.2
**Owning of Mobile**	No	17,280	61.0	612	3.8	8,120	50.5	7,342	45.7
	Yes	11,091	39.0	773	7.1	7,875	72.0	2,289‬	20.9

### Level of HIV-related stigma among sexually active Ethiopians

The study has depicted that only 5.1% (95% CI: 4.5%, 5.8%) of sexually active Ethiopians did not stigmatize PLWHA, whereas, 59.2% (95% CI: 57.3%, 61.1%) and 35.7% (95% CI: 33.5%, 37.9%) of them had shown moderate and high levels of stigmatized attitude towards PLWHA respectively (Table 2).

The study has also outlined HIV-related stigma in six different domains. More than four in eleven (36.7%) of the respondents manifested fear of HIV infection. More than one-third of the respondents had shown social judgment towards PLWHA. Close to three-fourth (72.7%) and about three in five (60%) of the respondents manifested anticipated and perceived stigma towards PLWHA. Nearly half (52.3%) of the respondents experienced stigma; whereas close to four in nine (43.2%) of the respondents were involved in discrimination (Table 3).

**Table 3:** Level of HIV-related stigma among sexually active Ethiopians, EDHS 2016

			**Total**		**Stigma level**					
					**No**		**Moderate**		**High**	
					5.1%^a^ (4.5%, 5.8%)		59.2%^a^ (57.3%, 61.1%)		35.7%^a^ (33.5%, 37.9%)	
			N	%	N	%	N	%	N	%
**Domains of Stigma**	**Fear of infection**	**No**	16,227	63.3	1,242	7.7	12,183	75.1	2,802	17.3
		**Yes**	9,422	36.7	0	0.0	2,990	31.7	6,432	68.3
	**Social judgment**	**No**	16,177	61.4	1,339	8.3	12,537	77.5	2,300	14.2
		**Yes**	10,190	38.6	0	0.0	2,985	29.3	7,205	70.7
	**Anticipated stigma**	**No**	6,893	27.3	1,126	16.3	4,628	67.1	1,140	16.5
		**Yes**	18,382	72.7	0	0.0	10,309	56.1	8,073	43.9
	**Perceived stigma**	**No**	10,261	39.3	1,229	12.0	7,171	69.9	1,861	18.1
		**Yes**	15,874	60.7	0	0.0	8,238	51.9	7,637	48.1
	**Experienced stigma (outside legal purview)**	**No**	12,665	47.7	1,334	10.5	10,207	80.6	1,124	8.9
		**Yes**	13,862	52.3	0	0.0	5,417	39.1	8,445	60.9
	**Discrimination (inside legal purview)**	**No**	14,926	56.8	1,307	8.8	11,442	76.7	2,177	14.6
		**Yes**	11,358	43.2	0	0.0	4,068	35.8	7,290	64.2

^a^ Indicates estimated percentage for each level of stigma and numbers in the bracket indicate their 95% confidence interval

### Results from a multilevel ordinal logistic regression model

In the process of model fitting, we have started with all candidate predictors included in the initial model and then applied backward elimination techniques to arrive at the final model. So, the results from the final model are presented as follows. To begin with the regional variability regarding stigmatization, the intraclass correlation among regions, show that the regional variability of the prevalence of HIV-related stigma was about 4%.

The odds of exercising a high level of HIV stigma instead of a low or moderate level, for rural dwellers compared to the urban is higher, adjusted odds ratio (AOR) = 1.82 with 95% CI [1.46, 2.27]. Regarding education, the odds of exercising a high level of HIV stigma were estimated to be AOR = 0.65 with 95% CI [0.51, 0.84] for primary, AOR = 0.42 with 95% CI [0.32, 0.56] for secondary and AOR = 0.43 to 95% CI [0.35, 0.54] for a higher level of education. For mobile telephone owners, the odds of showing a high level of stigma was lower compared to a participant without a mobile telephone, AOR = 0.63 with 95% CI [0.55, 0.72]. Likewise, for respondents who have ever tested for HIV/AIDS, the odds of showing a high level of stigma was lower compared to those who didn’t test and is estimated to be AOR = 0.77 with 95% CI [0.70, 0.84]. The odds of manifesting a high level of stigma instead of a low or moderate level, for late adults (aged 45–59 years), compared to youth (aged 15–29 years) was estimated to be AOR = 0.92 with 95% CI [0.84, 1.01].

For Protestant religion followers, the odds of exercising a high level of stigma instead of the low or moderate level, compared to Orthodox religion followers, was estimated to be AOR = 1.53 with 95% CI [1.33, 1.76]. The odds of manifesting a high level of stigma instead of a low or moderate level of stigma was estimated to be AOR = 1.38 with 95% CI [1.19, 1.61] for married respondents compared to those who were never married (Table 3).

## Discussion

The study has depicted that only 5.1 percent of sexually active Ethiopians did not have a stigmatized attitude towards PLWH, whereas, 59.2 percent and 35.7 percent of them had shown moderate and high levels of stigmatized attitude towards PLWH respectively. The study has shown that more than nine in ten (95%) of sexually active Ethiopians do have a stigmatized attitude towards people leaving with HIV which is by far higher than the results from the study done in Nigeria where only 50% of men and women aged 15–49 years had stigmatized attitude towards people living with HIV [29]. The result from the current study is also almost double and more than double of the result from the study done in the Heilongjiang Province of China; where 49.6% and 37.0% of the rural and urban resident men and women aged 15–69 years had stigmatized attitude towards PLWHA [30]. The differences in stigmatized attitudes towards PLWHA among sexually active Ethiopians and the other sexually active people residing in developed countries may be accounted for by the differences in access to media and education. The current study and many other studies have shown that more educated people and people who have more access to media have less stigmatized attitudes towards PLWHA [29–31].

The regional variability of the prevalence of levels of stigmatizing among sexually active men and women in Ethiopia towards PLWHA is close to 4%, which was not undermined and captured in the analysis. This could be explained by the difference in access to mass media and education in the different regions of the country. The 2016 Ethiopian demographic and health survey report depicted that there was a great disparity in the level of education attained among the inhabitants living in the different regions of the country. The report also showed that access to mass media was highly influenced by the level of literacy of the inhabitants [32]. On the other hand, the regional variation in the level of stigmatized attitudes towards PLWHA may be related to the disparities in the knowledge about HIV across the different regions. Different studies done in Ethiopia showed that the distribution of HIV knowledge significantly varies across the different regions of the country [33, 34]. The study done in Botswana depicted that HIV-related knowledge and stigma towards PLWHA are positively correlated [35].

The residence of the respondents was found to have a statistically significant association with levels of HIV/AIDS stigma. The odds of exercising a high level of HIV stigma instead of a low to moderate level of stigma towards PLWHA, for rural dwellers, was greater by 82% compared to urban dwellers, controlling for other factors included in the model. This finding is similar to the studies in Ethiopia that revealed that urban dwellers were knowledgeable about HIV/AIDS than rural residents and hence stigmatization is more common among rural than urban people [13, 36]. The finding from the current study is also consistent with the result of the study done in the Heilongjiang Province of China where urban dwellers had a less stigmatized attitude towards PLWHA as compared to rural dwellers [30]. On the contrary, the place of residence was not associated with the level of HIV stigma in the study conducted in Cameroon [37]. Most likely, this could be due to the reason that urban dwellers have more access to information than rural dwellers that can change their attitude towards PLWHA than those from rural areas. The insignificance of the place of residence in the study conducted in Cameroon might be related to the uniformity of the knowledge about HIV between the urban and rural, which is not the case in Ethiopia.

Regarding education, the result shows that it has a significant association with the level of HIV/AIDS stigma towards PLWHA. For participants who attended primary school compared to those who have never been to school, the odds of exercising a high level of HIV stigma instead of a low to moderate level of stigma towards PLWHA was lower by about 35%, holding the other factors in the model constant. Likewise, for those who have attended secondary and higher education, the odds of exercising a high level of HIV stigma instead of a low to moderate level of stigma were lower by 68% and 67% respectively, compared to participants who have never gone to school, ‘*ceteris paribus*’. This synthesized evidence is concordant with the studies done in Ethiopia, South Africa, and Cameroon in which level of education makes a difference on having stigmatizing level towards PLWHA [29, 30, 36–39]. Since education is a means of gaining knowledge about the problem and it is a means of any changes including reducing exercising stigma.

Mobile telephone owners are expected to have higher access to information compared to non-owners. Therefore, they may not be in a position to stigmatize PLWHA as they get more information about the way of transmission of the disease. This study revealed that owning or not wowing mobile telephones has a statistically significant association with the level of HIV stigma and those who own mobile telephones are less likely to manifest a higher level of HIV stigma towards PLWHA. This finding is supported by the existing evidence sorted out from EDHS 2011 reflected that frequent access to media had a lower tendency to stigmatize PLWHA [36] and hence owning a mobile telephone is a means of accessing media and information. The finding from the current study is also in line with the finding from the cross-sectional study done in sub-Saharan Africa where media use was associated with a low level of HIV-related stigma (Bekalu MA et at., 2014) [31].

The participants' status of test for HIV/AIDS was found to have a statistically significant relationship with the level of HIV stigma exercised. That means those who have ever tested for HIV/AIDS are very likely to get HIV/AIDS voluntary counseling and testing service. The current study revealed that, for those who have ever tested for HIV/AIDS, the odds of showing a high level of stigma instead of a low to moderate level of HIV stigma was lower by about 23%, controlling for the effect of other predictors in the model. This finding is in line with different studies done and identified HIV stigma as a factor that hinders the readiness of people to get tested [40, 41]. The similarities can be thought of as complementary to each other that the current study is the summaries of findings conducted in the narrower area of Ethiopia.

Religion was one of the factors significantly associated with a high level of HIV stigma. For Protestant religion followers, the odds of showing a high level of stigma instead of the low to moderate level of stigma was higher by about 53% compared to Orthodox religion followers. Although our study presented the details of the association between religion and level of stigmatization, we did not find any literature presenting results the same way and other studies just presented the association between religion and HIV-related stigma. For instance, the studies done in South Carolina on African-American face-based congregants and in Puerto Rico showed that being religious is highly associated with HIV-related stigma [42, 43]. Other studies done in Benin, Nigeria, and Tanzania also showed that religious people perceive HIV as a disease of sinners or people with lower moral standards and the punishment inflicted by God [44, 45]. This can be explained by HIV-related beliefs and dogmas of religious organizations.

Married respondents were more likely to manifest a high level of stigma instead of low to moderate levels of stigma compared to those who were never married. The pieces of evidence from the literature did not present the relationship between marital status and levels of stigmatization but generally showed that the association is significant. For example, finding this study is consistent with the findings of studies done in Nigeria and Tajikistan where married women had a more stigmatized attitude towards PLWHA [46, 47]. This could be due to the reason that people in a marriage union, most of the time, link sexually transmitted infection to unfaithfulness and lack of commitment to fidelity which makes them less tolerant to PLWHA [48, 49].

Age was found to have a significant association with the level of stigma towards PLWHA. Late adults, 45–59 years, were less likely to manifest a high level of stigma instead of a low to moderate level of stigma compared to the youth, 15–29 years. This result is concordant with the study from Thailand using nationally representative data where people in the age group of 20–39 years were 1.23 times more likely to have a stigmatized attitude towards PLWHA as compared to people in the age group of 40–49 years [50]. The study was done in rural China also showed that advancement in age is inversely proportional to the level of HIV-related stigma and a higher level of HIV-related stigma was observed among youths [12]. This might be due to different reasons. As people advance in age, their awareness of HIV/AIDS increases as a result of exposure to different media and advancement in educational level which promotes HIV-related knowledge and understandings. The current study and many other studies also depicted that advancement in educational level is inversely proportional to the level of HIV-related stigma [8, 42, 51, 52].

## Conclusion

The current study indicated that a high proportion of sexually active Ethiopians have yet stigmatized attitudes towards PLWHA regardless of all efforts taken to prevent and control the disease. As the study indicated, nearly 95% of sexually active Ethiopians have moderate to a high level of stigmatized attitude towards PLWHA. The study has also shown that the level of stigma attached to HIV/AIDS is extremely varied across the regions of the country. The results of multilevel ordinal logistic regression have shown that residence, educational level, owning mobile, ever test uptake, age, religion, and marital status were determinants of HIV-related stigma. Expanding mobile coverage, promoting HIV counseling and testing, promoting HIV education, and working with religious leaders are among other strategies that can be used to minimize the stigma attached to the disease to best prevent and control it. Future researchers interested in the area should also address the role of socio-cultural impact on HIV-related stigma.

### Strengths and limitations of this study

The sampling method used is to the standard as the nationally representative data were collected by the MEASUREDHS program. The data were weighted before doing analyses and multilevel modeling was also applied to account for the variation of levels of HIV-related stigma across the regions in Ethiopia. This study used data from a single time survey, the temporality between HIV-related stigma and the factors included here alone cannot be ascertained and the shreds of evidence should be utilized with care. Due to the lack of qualitative data on the 2016 EDHS, the authors were unable to investigate the association between some qualitative variables like socio-cultural factors and HIV-related stigma level; consequently, this study is limited to variables on EDHS.

## Data Availability

The dataset authors used is available on the following repository: https://dhsprogram.com/Where-We-Work/Country-Main.cfm?ctry_id=65&c=Ethiopia

## Abbreviations

AIC: Akaike Information Criterion
AIDS: Acquired immune deficiency syndrome
AOR: Adjusted Odds Ratio
ART: Anti-Retroviral Therapy
BIC: Bayesian Information Criterion
CI: Confidence Interval
CSA: Central Statistical Agency
EA: Enumeration Area
EDHS: Ethiopian Demographic and Health Survey
HIV: Human Immuno Virus
ICC: Intraclass correlation
PLWHA: People living with HIV AIDS
PPS: Probability Proportionate to Size
UNAIDS: United Nations Program on HIV/AIDS
VCT: Voluntary Counseling and Testing
WHO: World Health Organization
